# Magnetic Resonance Imaging Features and Prognostic Indicators of Local Recurrence after Curettage and Cementation of Atypical Cartilaginous Tumour in the Appendicular Skeleton

**DOI:** 10.3390/jcm12216905

**Published:** 2023-11-02

**Authors:** Amir Gahanbani Ardakani, Rebecca Morgan, George Matheron, Helard Havard, Michael Khoo, Asif Saifuddin, Panagiotis Gikas

**Affiliations:** 1 Department of Orthopaedic Oncology, Royal National Orthopaedic Hospital, London HA7 4LP, UK; 2 Department of Radiology, Royal National Orthopaedic Hospital, London HA7 4LP, UK

**Keywords:** chondrosarcoma, neoplasms, connective, soft tissue and bone, musculoskeletal diseases, local recurrence, MRI

## Abstract

**Objective**: The aim of this study is to determine MRI features that may be prognostic indicators of local recurrence (LR) in patients treated with curettage and cementation of atypical cartilaginous tumours (ACTs) in the appendicular skeleton. **Materials and Methods**: This study is a retrospective review of adult patients with histologically confirmed appendicular ACT. The data collected included age, sex, skeletal location and histology from curettage, the presence of LR and oncological outcomes. The pre-operative MRI characteristics of the ACT reviewed by a specialist MSK radiologist included lesion location, lesion length, degree of medullary filling, bone expansion, cortical status and the presence of soft tissue extension. **Results**: A total of 43 patients were included, including 9 males and 34 females with a mean age of 42.8 years (range: 25–76 years). Tumours were located in the femur (n = 19), humerus (n = 15), tibia (n = 5), fibula (n = 2) and radius and ulna (n = 1 each). A total of 19 lesions were located in the diaphysis, 12 in the metadiaphysis, 6 in the metaphysis and 6 in the epiphysis. The mean tumour length was 61.0 mm (range: 12–134 mm). The mean follow up was 97.7 months (range: 20–157 months), during which 10 (23.3%) patients developed LR, 7 (70%) of which were asymptomatic and 3 (30%) of which presented with pain. Four patients required repeat surgery with no associated death or evidence of metastatic disease. LR was significantly commoner with tumours arising in the epiphysis or metadiaphysis, but no MRI features were predictive of LR. **Conclusions**: No relationship was found between the apparent ‘aggressiveness’ of an ACT of the appendicular skeleton on MRI and the development of LR following treatment with curettage and cementation.

## 1. Introduction

Knowledge on the chondrosarcoma (CS) of bone has been largely based on single institutional retrospective reports on outcomes, which can vary greatly depending upon the surgical technique and histological interpretation [1]. CSs account for 20–30% of all malignant bone tumours, of which conventional central CS makes up the largest sub-group [2]. Central CS is a tumour that has a peak incidence in the 4th–7th decades of life [3]. Primary CS arises de novo from previously normal bone and tends to be located centrally, while secondary CS arises from a benign precursor lesion, most commonly the cartilage cap of an osteochondroma (peripheral CS) [4]. CS is a cartilage-forming malignant bone tumour and can be graded based on the degree of hypercellularity, pleomorphism, hyperchromatism, and cellular and nuclear atypia [5,6]. Histologically, CS is sub-classified as low-grade CS (atypical cartilaginous tumour (ACT)) when involving the appendicular skeleton, grade 1 CS when involving the flat bones and axial skeleton, high-grade CS (HG-CS; grades 2 and 3 CS) and dedifferentiated CS (DD-CS) [7].

The optimal treatment and subsequent outcome of CS are largely dependent upon its histological grade [8,9]. High-grade and dedifferentiated CS require wide local excision (WLE) with or without reconstruction, usually with an endoprosthetic replacement (EPR), while ACT/grade 1 CS is usually treated with intra-lesional curettage (IC) with or without various adjuvant treatments, in addition to bone grafting or cementation [8,9].

The roles of radiotherapy and chemotherapy are limited in the management of CS, and therefore, the mainstay of treatment is surgical resection/curettage [10]. The adequacy of surgical resection, often with higher-grade tumours, has shown to have a positive impact on both the prognosis and rate of recurrence [11]. Until recently, there has been debate with regard to the surgical treatment of low-grade CS, with some authors advocating for WLE, necessitating substantial reconstructive surgery, whilst others believe a more conservative surgical approach is warranted [3,12,13,14].

Studies describing the radiological prognostic factors that might contribute to LR are limited. It is suggested that local Recurrence (LR) tends to occur in the setting of a more aggressive lesion, and therefore, the diagnostic approach to central low-grade CS must be multidisciplinary, requiring a skilled team of radiologists, pathologists, oncologists and surgeons. The correct diagnosis will ultimately lead to suitable treatment and better outcomes [12,15]. The literature has described some MRI features that potentially differentiate a low-grade CS from a high-grade CS, such as a soft tissue mass being indicative of a high-grade lesion, whereas the presence of entrapped fat within the tumoural tissue is consistent with low-grade tumours [16,17]. No studies to date have specifically detailed the radiological prognostic indicators of LR in a biopsy-proven ACT, nor have they expanded on the importance and significance of the radiological detection of LR during follow-up.

Establishing knowledge of potential prognostic MRI features of LR would be useful and may, in turn, dictate and help with treatment decisions. The aim of the current study was to determine whether there are any prognostic indicators on pre-operative MRI for the development of LR that may aid in the effective management of these lesions.

## 2. Materials and Methods

This study was approved by the local Research and Innovation Centre of The Institute of Orthopaedics under the Integrated Research Application System number 262826, with no requirement for informed patient consent.

This was a retrospective study of all adult patients with a histologically proven diagnosis of ACT in the appendicular skeleton treated with curettage and cementation between January 2007 and January 2020. This yielded 75 patients in total, of whom 32 were excluded due to a lack of post-treatment imaging as a follow-up was completed elsewhere. This left 43 patients with an ACT for whom complete clinical information and imaging studies were available.

All patients had pre-operative MRI studies available for review, either from the referring hospital (n = 25) or performed at our centre following referral (n = 18; 14 at 1.5T and 4 at 3T). A Consultant Musculoskeletal Radiologists with 8 years of experience in tumour imaging reviewed all the MRI studies. The following criteria were assessed: lesion location, lesion length, the degree of medullary filling, bone expansion, cortical status and the presence of soft tissue extension.

All 43 patients underwent a surgical procedure in the form of curettage, high-speed burring and cementation alone, without the use of adjuvants. Operations were carried out by five different surgeons, each of whom specialise in sarcoma surgery. Sarcoma consultants where present at the time of each operation. The final histological grade of CS was therefore based on surgical specimens in all cases, which were routinely reported for clinical purposes by two specialist consultant histopathologists. Data were also collected on patient demographics, length of follow-up, evidence of LR clinically and on imaging and overall patient survival.

### Statistical Methods

All analyses examined the association between the various demographic and MRI factors and the presence of LR. The majority of the demographic and MRI variables were measured on a categorical scale, and therefore, the associations between these factors and LR were assessed using Fisher’s exact test. The exception was for lesion length, where an unpaired *t*-test was used to compare tumour length in patients with and without LR.

## 3. Results

Of the 43 patients, 9 (20.9%) were males and 34 (79.1%) were females with a mean age of 42.8 years (range: 25–76 years). LR was less common in males, but this did not quite reach statistical significance (*p* = 0.09). Tumours were located in the femur in 19 (44.2%) cases, humerus in 15 (34.9%) cases, tibia in 5 (11.6%) cases, fibula in 2 (4.7%) cases and radius and ulna in 1 (2.3%) case each.

Clinical findings: The mean follow-up was 97.7 months (range: 20–157 months). During this time, 10 (23.3%) patients developed a LR, and 7 (70%) were asymptomatic and detected on surveillance imaging alone. Of these, six (85.7%) were treated conservatively with surveillance alone and one (14.3%) underwent re-curettage and cementation with no further recurrence after 96 months. Three patients had symptomatic LR and all underwent further surgical treatment, with one having a WLE and endoprosthesis and two having revision curettage and cementation, which subsequently recurred. Eventually, both were treated with a WLE and endoprosthesis. All patients with LR were still alive at the last follow-up with no evidence of metastatic disease. Only one death was noted in the cohort of 43 patients (2.3%), and on review of the histology, this was likely to be an HGCT as opposed to the originally perceived ACT. The death of this patient was not tumour-related, and there was no evidence of metastatic disease at follow-up.

**Figure 1:**
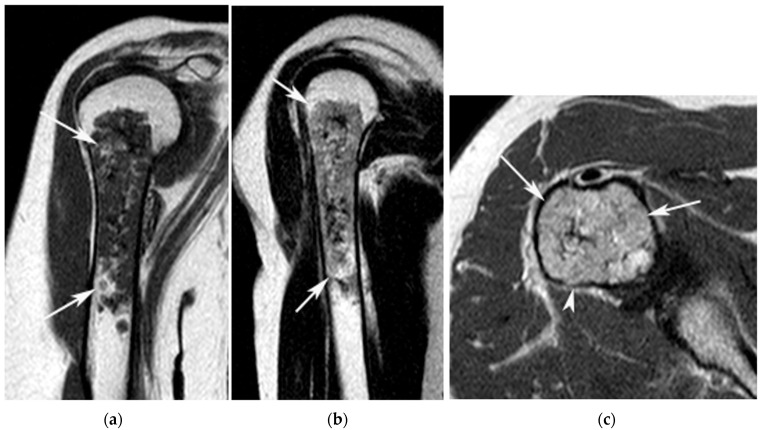
A 58-year-old female with an ACT in the right proximal humerus. (**a**) Coronal T1W TSE, (**b**) sagittal T2W FSE and (**c**) axial PDW FSE MR images show an extensive chondral tumour (arrows) centred on the proximal metaphyseal region of the humerus. The lesion fills the medullary cavity but does not result in any bone expansion or cortical destruction.

**Figure 2:**
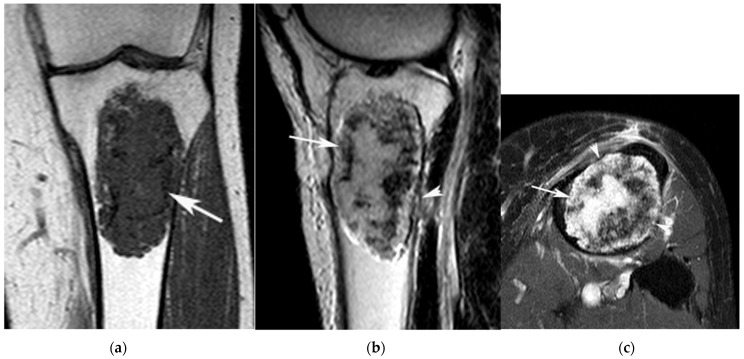
A 56-year-old female with an ACT in the left proximal tibia. (**a**) Coronal T1W TSE, (**b**) sagittal T2W FSE and (**c**) axial SPAIR MR images show a chondral tumour (arrows) centred on the proximal metaphysis of the tibia. The lesion fills the medullary cavity and results in multifocal deep endosteal scalloping (arrowheads (**b**,**c**)) but does not cause in any bone expansion or cortical destruction.

**Figure 3:**
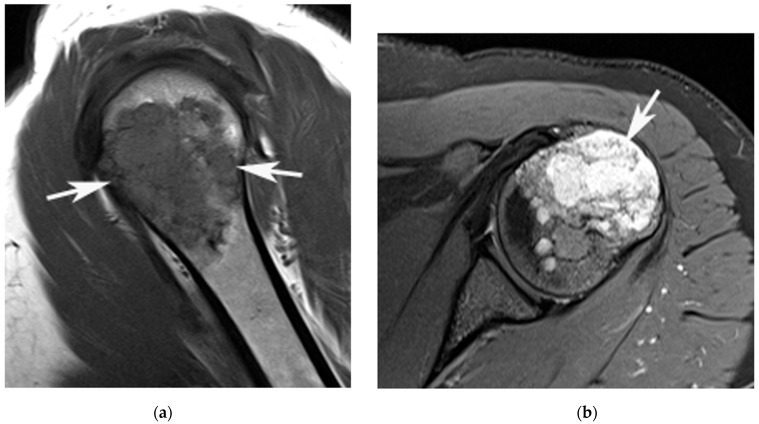
A 33-year-old female with an ACT in the left proximal humerus. (**a**) Sagittal T1W TSE and (**b**) axial SPAIR MR images show a chondral tumour (arrows) centred on the proximal metaphysis of the humerus. The lesion results in mild expansion of the lateral humeral cortex (arrow (**b**)) with no associated cortical breach.

**Figure 4:**
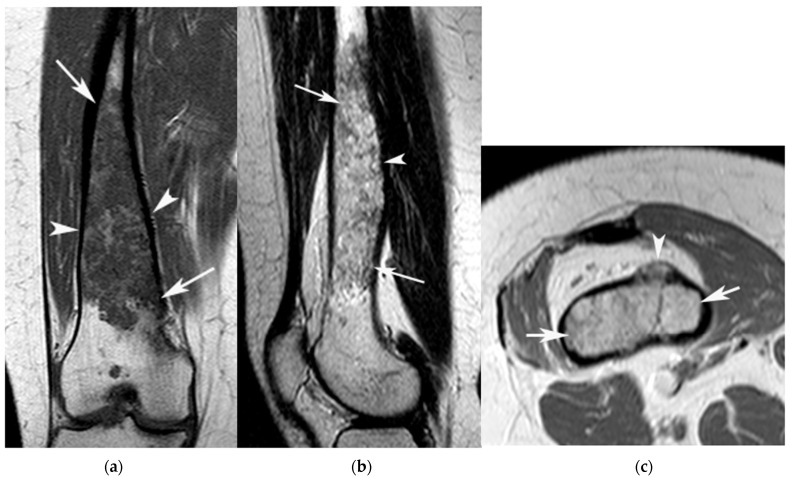
A 46-year-old female with Ollier disease and an ACT in the right distal femur. (**a**) Coronal T1W TSE, (**b**) sagittal T2W FSE and (**c**) axial PDW FSE MR images show an extensive chondral tumour (arrows) centred on the distal metametaphysis of the femur. The lesion fills the medullary cavity and results in bone expansion (arrowheads (**a**,**b**)). A small extra-osseous mass is also present (arrowhead (**c**)).

**Figure 5:**
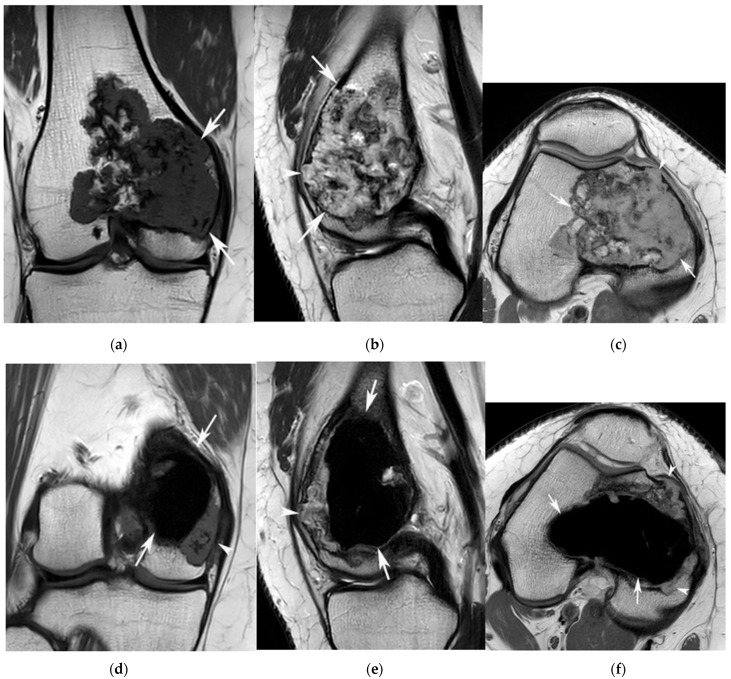
A 28-year-old female with an ACT in the right distal femur. (**a**) Coronal T1W TSE, (**b**) sagittal T2W FSE and (**c**) axial PDW FSE MR images show a lobular chondral tumour (arrows) extending into the epiphysis of the femur associated with areas of cortical breach (arrowheads (**b**,**c**)). Repeat imaging 9 months later. (**d**) Coronal T1W TSE, (**e**) sagittal T2W FSE and (**f**) axial PDW FSE MR images show the cementoma (arrows) following curettage and multifocal areas of local recurrence (arrowheads).

MRI findings: In total, 19 (44.2%) lesions were located in the diaphysis, 12 (27.9%) in the metadiaphysis, 6 (14%) in the metaphysis and 6 (14%) in the epiphysis. The mean maximal tumour length was 61.0 mm (range: 12–134 mm). The tumour filled the medullary cavity in 21 (48.8%) cases (Figure 1), caused cortical scalloping in 28 (65.1%) cases (Figure 2), resulted in bone expansion in 13 (30.2%) cases (Figure 3), breached the cortex in 6 (14%) cases and was associated with soft tissue extension in 1 case (Figure 4). The relationship between MRI features and LR is presented in Table 1. This demonstrates that LR was significantly commoner in the epiphysis (50%) (Figure 5) and metadiaphysis (42%), whilst LR occurred in 25% or less patients at the other two locations. No other MRI features were predictive of LR.

## 4. Discussion

The aim of the current study was to identify features on pre-operative MRI that may be predictive of LR in patients with ACT of the appendicular skeleton. Although it might have been expected that more aggressive imaging features, such as a tumour filling the medullary cavity, bone expansion, cortical thinning, cortical breach and soft tissue mass, would be associated with LR, this was not the case. The only feature that showed a significant difference between those with and without LR was tumour location in the epiphysis or metadiaphysis. These findings would therefore support the use of curettage and cementation for biopsy-proven ACT of the major long bones irrespective of the apparent ‘aggressiveness’ of the tumour on MRI.

Care must be taken to neither overtreat benign tumours nor undertreat malignant ones. The principles of surgical treatment for CS balance reducing oncological risk (recurrence and metastasis) whilst maximizing functional outcome and minimizing the damage caused by more radical surgical techniques [18]. The literature now advocates for the use of IC for the treatment of low-grade chondral lesions. Chen et al. undertook a meta-analysis of 10 studies looking at over 390 patients surgically treated for low-grade chondral lesions with follow-ups of over 2 years [19]. The study found no significant difference in outcomes between IC and WLE. IC was associated with fewer complications and better functional outcome scores without increasing the risk for LR or metastases [20]. The study went on to demonstrate that the risk of LR was not influenced by the use of adjuvant therapies, and failed to demonstrate superiority of one adjuvant over another or when compared to no adjuvant use [19]. More recently, Shemesh et al. published a series on the surgical management of 32 patients with a low-grade CS with a 10-year follow-up [20]. They demonstrated excellent local control with IC with only one case of LR and no cases of metastatic disease. They found their complication rates to be low and functional scores to be high. Similarly, they found no significant oncological advantage to WLE, but they did find an increase in complications and a reduction in functional scores with the latter [20]. These findings are echoed by several other authors and reinforce the shift to a more surgically conservative approach in a correctly diagnosed low-grade chondral tumour of the appendicular skeleton [3,5,9,19,20,21,22,23].

The potential for LR and metastases from ACT is extremely low, with reported 5-year survival rates ranging from 85 to 100% following various treatment strategies [22]. Given that the literature has shown no statistically significant advantage to WLE in terms of LR or metastases, the focus moved to determining the oncological benefit of the additional use of adjuvants for IC [9]. The published data suggest that the rate of LR can range from 0 to 13.3% in patients with ACTs of long bones treated with IC, with or without various adjuvant treatments, followed by bone grafting or cementation. Cementation itself, as well as providing structural support, can also be seen as an oncological adjuvant [21]. Adjuvant therapy comes in the form of chemical adjuvants such as phenol and alcohol, cryosurgery, high-speed burring and the use of the thermogenic effect of bone cement. Phenol and cryotherapy are the two most common adjuncts and have a LRs of 3.1–13.3% and 0–9.3%, respectively [21]. A series of 24 patients who had undergone manual IC with a high-speed burr and cementation alone reported excellent results with no LR at a median follow-up of 66 months [21]. Other published studies have also reported good oncological outcomes (LR 0–5%) with mechanical curettage alone and the thermogenic effect during the polymerization of bone cement [3,22]. The use of adjuvant therapy is also hindered by its associated complications. Phenol can cause chemical burns, and cryotherapy can be technically demanding, with some studies suggesting the potential to weaken the adjacent bone and cause pathological fracture [3].

Shemesh et al. published a meta-analysis of the data surrounding treatment strategies for central low-grade CS of long bones. They found the data on adjuvant therapy with IC to be inconsistent, with multiple adjuvant therapies being used in the same patient. This made it very difficult to appraise the individual adjuvant treatments and make judgments on the oncological outcomes. Their analysis showed no statistical differences between the adjuvant modalities with regard to LR rates, complication rates, or re-operation rates [9].

Although our LR was found to be on the upper end (23%) of the LR rates reported in the literature, our follow-up times where much longer, our histology was consistent and accurate across the patient cohort included, and the majority of recurrences were picked up on imaging alone (70%) without any clinical symptoms. The large variability in the reported LR rates, as well as the differences in the reported impact of LRs on patient survival, raises fundamental questions regarding prognostic indicators, follow-up and surveillance methods [9]. Previous studies have described a negative impact on survival as a result of LR. Work completed by Schwab et al. found that the LR of Grade 1 chondrosarcoma in the long bones of extremities was associated with substantially worse overall survival when compared with patients without LR [24]. Although the study had limitations, they concluded that LR could signify a more aggressive tumour phenotype, and therefore advocated for the practice of treating LR more aggressively with WLE when possible. This is consistent with the current study, where two patients developed further recurrence after revision IC.

Disputing this, most other studies have suggested that the development of systemic disease is independent of LR [25]. A meta-analysis by Kim et al. found a post-LR survival rate of 59% at 10-years, suggesting that the development of LR does not necessarily correspond with poor survival. This is in keeping with data collected by other studies and this current study, which demonstrated a survival rate of 100% following the development of LR [26,27]. However, they also concluded that LR tends to be indicative of more aggressive biology, especially in those with older age at the onset of LR and a shorter interval from primary surgery. They support the opinion that wide surgical margins at LR surgery should be considered as this reduces the risk of subsequent LR [25].

LR can also be very difficult to distinguish from residual disease. A recent study by Dierselhuis et al. retrospectively reviewed low-grade CS treated surgically with IC and found that residual tumours have no impact on patient survival, since neither actual LR nor the upgrading of tumour histology occurred in the residual tissue [9,28].

We also note that studies suggesting LR may be indicative of more histologically aggressive biology. Our radiological data did not find any significant correlation between MRI features of ‘aggressiveness’ and the rate of LR. However, this study did find that the location of an ACT is an important and significant predictor of recurrence. LR was significantly higher for primary epiphyseal (50%) and metadiaphyseal ACTs (42%) when compared to diaphyseal lesions. The literature has often identified the epiphysis to be associated with a higher rate of recurrence, especially at the articular surface [29]. One hypothesis for this could be that it is due to the reluctance of the surgeon to damage this region, leading to less aggressive curettage, burr evasion and less electrocautery [30]. The epiphysis/metadiaphysis of long bones also have a more complex 3D geometric structures when compared to the simple tubular morphology of the diaphysis, such that the limitations of curette shapes and anatomical access may preclude more complete surgical curettage. The notion that the physeal status is a factor associated with aggressive tumour biology is controversial and has not been proven. Therefore, it is unlikely to be the cause of higher recurrence rates [31]. This would be in keeping with our findings.

## 5. Limitations

The current study has several limitations. Firstly, due to its retrospective nature, there were many patients who underwent follow-up elsewhere, and we therefore did not have post-treatment imaging for all patients. However, there is nothing to suggest that this resulted in bias towards patients who were more or less likely to have LR. The MRI studies were reviewed by a single, albeit experienced, MSK tumour radiologist; the MRI features of appendicular ACTs are well described and it is not felt that the addition of a second reader would have significantly changed the results. The patient group chosen were those for whom a decision based on available clinical, imaging and biopsy findings resulted in a surgical decision to undertake IC. Therefore, no comparison can be made with patients who had the same diagnosis for which a decision had been made to undertake primary WLE and endoprosthetic replacement. However, the results do indicate that when a decision is made based on available information to undertake IC, this is correct in the vast majority of cases irrespective of MRI appearance.

We also note the small sample size of the complete data, which may have influenced the absence of statistically significant associations between local recurrence and the aggressiveness of MRI findings. Conducting a power calculation for sample size indicates that doubling the number of patient subjects could decrease the likelihood of Type I and II errors. Therefore, future research endeavours should focus on analysing a larger sample size of patients to enhance the robustness of the findings.

## 6. Conclusions

The current study aimed to determine prognostic factors on pre-operative MRI that may be predictive of LR following curettage and cementation for ACT of the appendicular skeleton. LR was independent of the apparent ‘aggressiveness’ of the original tumour on MRI, but was significantly commoner for tumours arising in an epiphyseal or metadiaphyseal location. These findings suggest that IC and cementation is a safe option for treating biopsy-proven ACTs of the appendicular skeleton irrespective of the MRI appearance. It was also found that 70% of LRs were not associated with symptoms and that re-curettage of a symptomatic LR is likely to fail. The high rate of asymptomatic recurrence provides a strong argument for continued routine MRI surveillance after initial treatment.

## Figures and Tables

**Table 1:** Associations between various clinical and MRI features and local recurrence for 43 appendicular ACTs.

Variable	Category	No Recurrence Number (%)	Recurrence Number (%)	*p*-Value
Sex	Female	24 (71%)	10 (29%)	0.09
	Male	9 (20%)	0 (0%)	
Lesion location 1	Femur	15 (79%)	4 (21%)	0.49
	Humerus	10 (67%)	5 (33%)	
	Tibia	5 (100%)	0 (0%)	
	Minor long bones	3 (75%)	1 (25%)	
Lesion location 2	Diaphysis	18 (95%)	1 (25%)	**0.02**
	Epiphysis	3 (50%)	3 (50%)	
	Metadiaphysis	7 (58%)	5 (42%)	
	Metaphysis	5 (83%)	1 (17%)	
Lesion length (mm) ^(^*^)^	-	61.4 ± 28.0	63.3 ± 24.0	0.85
Medullary fillings	Complete	14 (67%)	7 (33%)	0.16
	Partial	19 (86%)	3 (14%)	
Bone expansion	No	24 (80%)	6 (20%)	0.46
	Yes	9 (69%)	4 (31%)	
Cortical status	Normal	8 (89%)	1 (11%)	0.66
	Scalloping/thinning	20 (71%)	8 (29%)	
	Cortical breach	5 (83%)	1 (17%)	
Soft Tissue mass	No	32 (76%)	10 (24%)	1.00
	Yes	1 (100%)	0 (0%)	

(*) Mean ± standard deviation for patients with/without recurrence reported. Analysis was performed using unpaired *t*-test.

## Data Availability

Not applicable.
